# Intermittent Fasting as a Neuroprotective Strategy: Gut–Brain Axis Modulation and Metabolic Reprogramming in Neurodegenerative Disorders

**DOI:** 10.3390/nu17142266

**Published:** 2025-07-09

**Authors:** Zaw Myo Hein, Muhammad Faqhrul Fahmy Arbain, Suresh Kumar, Muhammad Zulfadli Mehat, Hafizah Abdul Hamid, Muhammad Danial Che Ramli, Che Mohd Nasril Che Mohd Nassir

**Affiliations:** 1 Department of Basic Medical Sciences, College of Medicine, Ajman University, Ajman P.O. Box 346, United Arab Emirates; z.hein@ajman.ac.ae; 2 Department of Anatomy and Physiology, School of Basic Medical Sciences, Faculty of Medicine, University Sultan Zainal Abidin, Kuala Terengganu 20400, TGG, Malaysia; faqhrulfahmy@gmail.com; 3 Department of Diagnostic and Allied Health Science, Faculty of Health and Life Sciences, Management and Science University, Shah Alam 40100, SEL, Malaysia; sureshkumar@msu.edu.my; 4 Department of Human Anatomy, Faculty of Medicine and Health Sciences, Universiti Putra Malaysia, Serdang 43400, SEL, Malaysia; m_zulfadli@upm.edu.my (M.Z.M.); a_hafizah@upm.edu.my (H.A.H.); 5 Brain and Mental Health Research Advancement and Innovation Networks (PUTRA BRAIN), Faculty of Medicine and Health Sciences, Universiti Putra Malaysia, Serdang 43400, SEL, Malaysia

**Keywords:** intermittent fasting, gut–brain axis, neurodegenerative diseases, metabolic reprogramming, SCFAs, autophagy

## Abstract

Intermittent fasting (IF) is emerging as a heterogeneous neurometabolic intervention with the possibility of changing the course of neurodegenerative diseases. Through the modulation of the gut–brain axis (GBA), cellular bioenergetics (or metabolic) reprogramming, and involvement in preserved stress adaptation pathways, IF influences a range of physiological mechanisms, including mitobiogenesis, autophagy, circadian rhythm alignment, and neuroinflammation. This review critically synthesises current preclinical and early clinical evidence illustrating IF’s capability to supplement synaptic plasticity and integrity, reduce toxic proteins (proteotoxic) burden, and rehabilitate glial and immune homeostasis across models of Alzheimer’s disease, Parkinson’s disease, Huntington’s disease, and amyotrophic lateral sclerosis. The key players behind these effects are bioactive metabolites such as short-chain fatty acids (SCFA) and β-hydroxybutyrate (BHB), and molecular mediators such as brain-derived neurotrophic factor (BDNF). We feature the therapeutic pertinence of IF-induced changes in gut microbiota composition, immune response, and mitochondrial dynamics, and we discuss emerging approaches for merging IF into precision medicine frameworks. Crucial challenges include individual variability, protocol optimisation, safety in cognitively vulnerable populations, and the need for biomarker-guided, ethically grounded clinical trials. Finally, we propose IF as a scalable and flexible intervention that, when personalised and integrated with other modalities, may reframe neurodegeneration from a model of irreversible decline to one of modifiable resilience.

## 1. Introduction

Neurodegenerative diseases such as Alzheimer’s disease (AD), Parkinson’s disease (PD), Huntington’s disease (HD), and amyotrophic lateral sclerosis (ALS) represent a leading public health concern owing to their chronic progression, limited therapeutic options, and overwhelming impact on cognitive and motor functions [1,2]. The prevalence of these conditions is mounting with the ageing global population, putting a rising burden on healthcare systems and caregivers [3]. Regardless of substantial increases in understanding their pathology, existing therapeutic strategies mostly remain symptomatic, with limited options accessible to cease or reverse disease progression.

Recently, the gut–brain axis (GBA) has gained considerable interest as a potential modifiable factor in the development and progression of neurodegenerative diseases. The GBA incorporates a reciprocal communication network between the central nervous system (CNS) and the gastrointestinal tract, involving neural, metabolic, hormonal, and immunological pathways [4]. Disturbance of this axis through gut dysbiosis, impaired barrier function, or altered microbial metabolites has been associated with neuroinflammation, protein aggregation, and cognitive decline [5]. The gut microbiota, through the production of neuroactive metabolites like short-chain fatty acids (SCFA), can substantially impact neuronal signalling and brain health [6,7].

Corresponding to the mounting interest in the GBA, intermittent fasting (IF) has surfaced as a hopeful dietary strategy with sweeping effects on metabolism, immunity, and longevity [8]. IF comprises diverse eating patterns that alternate between periods of fasting and feeding, incorporating time-restricted feeding (daily eating limited to 6–10 h, aligned with circadian rhythm), alternate-day fasting (alternating 24-h periods of fasting and eating), and the 5:2 diet (i.e., five days of normal eating and two days of restricted calories) [9]. In this review the terms “IF” are reserved for generic discussion or comparisons across regimens. These routines have been demonstrated to trigger adaptive cellular stress responses, improve insulin sensitivity, and induce metabolic switching from glucose to lipid and ketone-based energy sources [10,11]. Of high relevance to neurodegenerative diseases, IF has been linked with improved mitochondrial efficiency [12], increased autophagy [13], decreased oxidative stress, and modulation of inflammatory response [14,15], all of which are implicated in the pathophysiology of cognitive and neurodegenerative disorders.

This review examines the crosstalk between IF, the GBA, and neurodegeneration. Exclusively, we explore how IF moderates gut microbiota composition, impacts key metabolic and signalling pathways, and confers neuroprotective effects throughout different neurodegenerative models. Through critical evaluation of preclinical and clinical evidence, we emphasise evolving mechanisms, recognise knowledge gaps, and discuss future directions for the clinical application of IF in dealing with neurodegenerative diseases.

## 2. IF and the GBA

### 2.1. Gut Microbiota and Roles of SCFA

The gut microbiota comprises trillions of microorganisms vital for host metabolism, immunity, and brain health, which have been extensively described in previous literature [16,17,18,19]. IF leads to significant restructuring of gut microbial communities, usually resulting in enhanced alpha diversity and increased taxa with anti-inflammatory and neuroactive characteristics [20]. For example, IF has been linked to higher levels of *Akkermansia muciniphila*, *Lactobacillus*, *Faecalibacterium prausnitzii*, and *Bifidobacterium longum*; bacteria recognised for producing beneficial metabolites and regulating immune responses [19,20].

Among these metabolites, SCFA, notably butyrate, propionate, and acetate, are fundamental signalling molecules in the GBA [21]. Whereby, recent studies demonstrate that IF not only increases microbial diversity but also enriches a broader spectrum of SCFA-producing bacteria beyond butyrate producers. For instance, *Eubacterium rectale*, *Roseburia* spp., and *Anaerostipes* spp. have been reported to increase following IF protocols and contribute to the biosynthesis of propionate and acetate [19,21]. Propionate modulates hepatic gluconeogenesis and engages free fatty acid receptors (FFAR2/3), influencing vagal afferent signalling and hypothalamic neuropeptide regulation. Moreover, acetate, the most abundant SCFA, plays key roles in appetite control, microglial maturation, and central energy homeostasis [22]. Moreover, IF enriches *Bacteroides* spp., which contribute to propionate production via the succinate pathway [21]. These microbial and functional shifts indicate that IF dynamically reprograms the gut ecosystem, potentially impacting CNS health through metabolite-mediated pathways. In addition, butyrate exerts neuroprotective effects by maintaining epithelial barrier integrity [22], ameliorating microglial activation [23], and upregulating the expression of neurotrophic factors, i.e., brain-derived neurotrophic factor (BDNF) [24]. Moreover, SCFAs have been shown to serve as epigenetic regulators through inhibition of histone deacetylase (HDAC), hence changing the transcriptional landscape in favour of synaptic plasticity and cognitive resilience [25].

Current preclinical studies have shown that IF-induced increases in SCFA levels are associated with increased hippocampal synaptic density [26] and decreased tau protein phosphorylation in AD models [27]. Other preclinical studies using the 3xTg and 5xFAD mouse model of AD have demonstrated that alternate day fasting positively modulates gut microbiota composition (i.e., *Bifidobacterium pseudolongum*), increasing the production of SCFA (i.e., butyrate, propionate, and acetate), which are known to support neuroplasticity [28,29]. This was supported by evidence from a systematic review that suggested that these metabolic changes may contribute to improved hippocampal long-term potentiation, hence better cognitive function [30]. Moreover, in human studies, time-restricted eating has been associated with elevated faecal SCFA concentrations, specifically in elderly people with mild cognitive impairment, implying translational importance [31].

**Figure 1:**
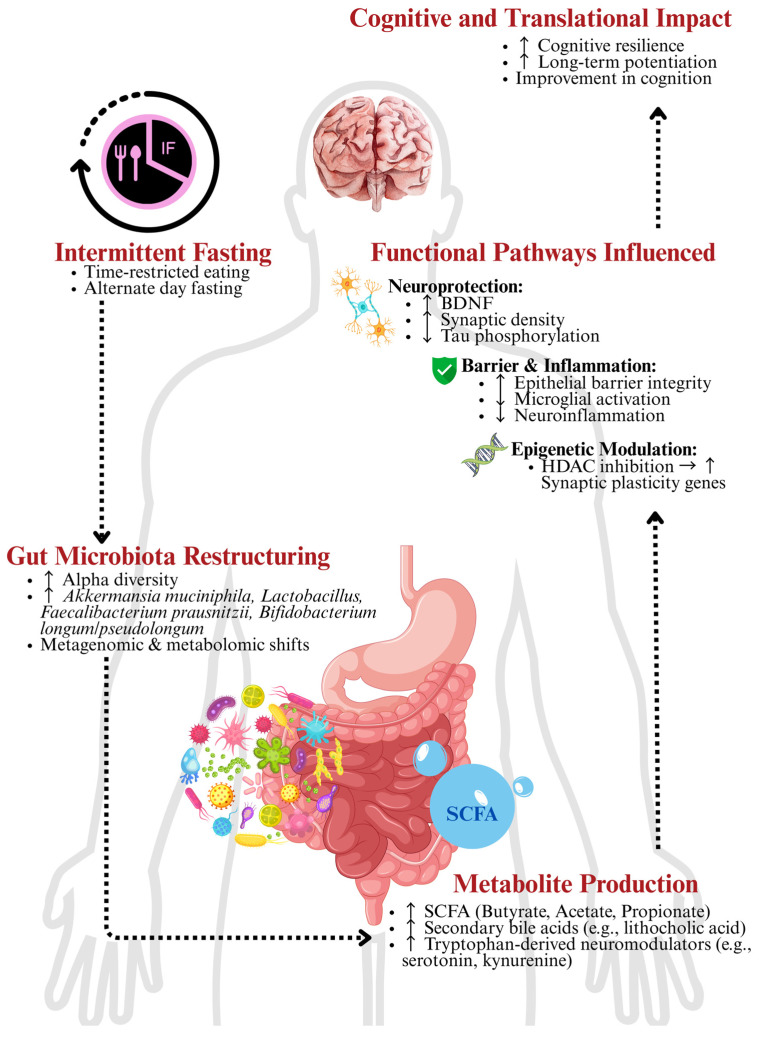
Gut–brain axis (GBA) pathways modulated by intermittent fasting (IF). Schematic diagram showing that IF alters the gut microbiota, increasing taxa associated with short-chain fatty acid (SCFA) production. SCFA, particularly butyrate, enhances gut barrier integrity, reduces neuroinflammation, and increases brain-derived neurotrophic factor (BDNF) expression via epigenetic mechanisms. These changes influence the GBA, improving hippocampal synaptic plasticity and cognitive function in neurodegenerative disease in both pre-clinical and clinical models. HDAC, histone deacetylase. Upward arrow (↑) indicates increase/heighten; downward arrow (↓) indicates decrease/reduce; forward arrow (→) indicates leading to/causing.

Moreover, the effect of IF on the gut microbiota extends beyond compositional changes, inducing metagenomic and metabolomic shifts that modulate host physiology. IF has been demonstrated to upregulate microbial gene expression associated with SCFA biosynthesis, particularly enhancing butyrate-producing taxa such as *Faecalibacterium prausnitzii* and *Eubacterium rectale* [32]. Additionally, IF modifies bile acid metabolism by promoting the conversion of primary to secondary bile acids like lithocholic acid, and modulates tryptophan pathways, increasing the production of neuromodulatory metabolites such as serotonin and kynurenine [33]. Hence, these findings feature the value of viewing the gut microbiome not only through taxonomic lenses but as a dynamic functional ecosystem responsive to dietary timing. Figure 1 summarises the interrelationship between IF and GBA.

### 2.2. Modulation of Neuro-Immuno-Inflammation

The gut microbiota is a key modulator of immune responses at both peripheral and central levels. Microbial metabolites, particularly SCFAs, exert anti-inflammatory effects by regulating cytokine production, enhancing epithelial integrity, and suppressing microglial activation [21,22,23,24,25]. Conversely, dysbiosis and microbial endotoxins such as lipopolysaccharide (LPS) can trigger systemic inflammation and crosstalk with brain-resident immune cells through the GBA. Moreover, IF is known to modulate this axis by enriching beneficial microbes, reducing endotoxin load, and increasing SCFA availability, thereby shaping the host’s inflammatory milieu.

Chronic low-grade inflammation and inflammaging (or age-related chronic, low-grade, and sterile inflammation) originating from the gut is progressively recognised as a significant mechanism in neurodegeneration [34,35]. One of the fundamental pathophysiological contributors is intestinal permeability, or “leaky gut,” which accelerates the translocation of microbial endotoxins such as lipopolysaccharides (LPS) into systemic circulation [36]. These endotoxins initiate pattern recognition receptors, specifically toll-like receptor 4 (TLR4), on immune cells and brain-resident microglia, initiating a cascade of pro-inflammatory cytokines involving interleukins such as interleukins (IL-1β and IL-6), and tumour necrosis factor alpha (TNF-α) [37,38]. However, through IF-induced enhancement of SCFA-producing microbes, epithelial integrity is improved, thereby limiting systemic exposure to endotoxins [39].

Available data have shown that IF can intersect this cycle via multiple converging mechanisms. First, IF enhances intestinal barrier integrity by upregulating the expression of tight junction proteins such as occludin and claudin-1, thereby preventing microbial translocation; this process has been linked to butyrate and propionate-producing microbes [39]. Second, SCFA, particularly butyrate, improve mucosal immunity and inhibit pro-inflammatory signalling by preventing nuclear factor-kappa B (NF-κB) activation [40] and NLRP3 (NOD-, LRR- and pyrin domain-containing protein 3) inflammasome assembly [41].

Thirdly, IF can moderate systemic inflammation; a meta-analysis of randomised controlled trials reported that IF schedules significantly mitigate C-reactive protein (CRP) levels, especially in overweight and obese individuals and with treatment durations of eight weeks or more [42]. However, the effects of IF on IL-6 levels are less consistent, with some studies reporting no significant changes [43]. Importantly, IF-mediated reduction in systemic IL-6 and CRP, where it is previously reported as elevated in AD, likely reflects decreased gut permeability and immune activation, although these markers may not be directly linked mechanistically. Therefore, it should be highlighted that such associations indicate correlation rather than causation.

Additionally, IF has been correlated with a decline in circulating monocytes, which are major players in the body’s inflammatory response. Research suggests that short-term fasting may reduce monocyte metabolic and inflammatory activity, leading to fewer circulating monocytes [44]. These effects are increasingly linked to microbial shifts under IF conditions, including reductions in LPS-producing Enterobacteriaceae and increases in SCFA-producers. Therefore, IF appears to reshape the systemic immune landscape by reducing pro-inflammatory markers like CRP and circulating monocyte levels. However, further research is needed to fully understand the mechanisms and to clarify the effects on other inflammatory markers such as IL-6.

Importantly, the anti-inflammatory effects of IF are not limited to peripheral immunity. A study by Rangan et al. [45] reported that IF can mitigate neuroinflammation and cognitive decline in neurodegenerative disorder, i.e., in AD models. In 3xTg-AD mice, cycles of a fasting-mimicking diet (FMD) reduced microglial density in the hippocampus and cortex, decreased expression of M1 markers (e.g., inducible nitric oxide synthase (iNOS) and CD86), and increased M2 markers (e.g., Arg1, IL-10) [45]. These changes were linked with improved cognitive performance and synaptic preservation, influenced in part by gut-derived signals such as butyrate, acetate, and indole derivatives.

Recent work has shown that IF-induced microbiota modulation leads to decreased pro-inflammatory gene expression in the hippocampus and cortex, independent of disease-specific pathology [46,47]. These findings indicate that microbiota–immune–brain interactions form a key axis of IF’s neuroprotective action, and not merely an adjunct effect. For example, a study by Wu et al. [46] reported that IF ameliorates Aβ deposition and cognitive impairment in an AD mouse model. The intervention decreased lipid droplet aggregation within microglia, enhancing their phagocytic activity and contributing to the clearance of amyloid plaques.

Furthermore, research by Whittaker et al. [47] demonstrated that time-restricted feeding improved memory and reduced Aβ accumulation in the brain of AD mice. The study implied that aligning feeding regimes with circadian rhythms could be a promising strategy for mitigating AD pathology. Together, these studies signify that IF not only modulates peripheral immunity but also employs significant anti-inflammatory effects within the CNS, suggesting a potential non-pharmacological approach to mitigate AD.

Emerging evidence also suggests that IF modulates gut-derived neurotransmitter pathways, including tryptophan and serotonin metabolism [33]. Improved microbial conversion of tryptophan to indole derivatives under IF conditions may provide neuroprotective effects via aryl hydrocarbon receptor (AhR) signalling and maintenance of intestinal–immune balance [48]. Table 1 summarises the key inflammatory mediators affected by IF in GBA, with associated signalling pathways and effects on neurodegeneration (e.g., TLR4, NF-κB, NLRP3, SCFA, cytokines).

### 2.3. Circadian Rhythm and Chrononutrition

The timing of nutrient intake (chrononutrition) is progressively acknowledged as a significant contributing factor to gut and brain function, especially in GBA. Circadian rhythms coordinate physiological mechanisms such as hormone secretion, immune surveillance, and microbial activity [49]. Disturbance of these rhythms has been linked with neurodegenerative diseases [50] and metabolic disorders [51]. IF, such as time-restricted feeding, has been shown to reinforce circadian alignment by synchronising feeding–fasting cycles with the body’s intrinsic molecular clocks. This synchronisation re-establishes rhythmicity in metabolic and immune pathways, many of which are controlled by the core clock genes (e.g., BMAL1, CLOCK, PER, CRY). In this context, IF improves circadian amplitude and phase coherence of key regulatory genes, contributing to optimal mitochondrial function, antioxidant enzyme expression, and inflammatory gene suppression [52,53]. Moreover, in the gut, IF-induced circadian alignment promotes diurnal oscillations in microbial composition and metabolite production. For instance, time-restricted feeding enhances the rhythmic abundance of SCFA-producing taxa such as *Butyricicoccus*, *Lactobacillus*, and *Faecalibacterium*, resulting in peak butyrate and propionate production during feeding phases, which supports gut barrier integrity and modulates neuroinflammatory tone [54]. These time-locked microbial signals also influence CNS function via vagal and humoral routes. In addition, TRF has also been associated with better sleep patterns, decreased hypothalamic inflammation, and improved neurogenesis, hence improving cognitive performance [47]. The current literature has also discussed that individuals sticking to an early time-restricted eating schedule (8 a.m. to 4 p.m.) demonstrated improved melatonin secretion, more robust cortisol rhythms, and sleep quality, factors that interact with glymphatic clearance and neurorestorative processes [55]. Such chronobiological improvements may be particularly relevant for elderly or cognitively impaired populations, where circadian desynchrony exacerbates neurodegenerative risk [55,56].

Additionally, neuroinflammation is also circadian-sensitive, whereby misaligned feeding schedules (e.g., nocturnal eating) elevate hypothalamic inflammation and microglial activation, while IF restores hypothalamic homeostasis, reduces lipocalin-2 expression, and enhances astrocytic clearance pathways [55,56]. In neurodegenerative models, time-restricted feeding has been shown to reduce hippocampal inflammation, enhance neurogenesis, and restore circadian expression of BDNF and clock-controlled mitochondrial genes, leading to improved memory and synaptic resilience [47,57,58,59].

IF’s impact on circadian rhythms may also modulate mitochondrial dynamics and redox homeostasis in the brain [57]. For example, Sirtuin 1 (SIRT1) protein encoded by the *SIRT1* gene, a main fasting-responsive gene, functions as a circadian modulator and upregulates neuroprotective genes, including BDNF and peroxisome proliferator-activated receptor-γ coactivator 1-α (PGC-1α) [58,59]. These links indicate that IF could serve as a potential chrono-therapeutic strategy, particularly in ageing populations with disrupted circadian rhythms, that is more prone to neurodegenerative diseases.

Thus, IF-mediated circadian alignment offers a multifaceted benefit including entraining microbial oscillations, dampening neuroinflammation, and activating neuroprotective gene networks. These mechanisms underscore the importance of not only “what” and “how much” we eat but also “when” we eat in shaping brain health and ageing trajectories. Moreover, their interrelationship features the GBA not just as a pathophysiological bridge, but as a fertile therapeutic target in the context of neurodegenerative disease.

Additionally, while the GBA is often described through the influence of microbial signals on brain function, it is important to recognise its bidirectional nature. In addition to gut-derived metabolites (e.g., SCFAs, tryptophan derivatives) influencing neural, immune, and endocrine pathways, the brain also regulates gut physiology through descending neural and hormonal signals. Hypothalamic centres, in response to feeding cues and circadian inputs, modulate vagal efferent activity that shapes gut motility, mucus secretion, and microbial habitat [49,50,51]. Furthermore, activation of the hypothalamic–pituitary–adrenal (HPA) axis under psychological or metabolic stress alters intestinal permeability and immune tone via glucocorticoids and autonomic nervous system signals. Studies have shown that vagal nerve stimulation, altered sleep–wake cycles, and mood disturbances can directly impact gut microbial composition and function [50,51]. Therefore, IF may influence the GBA not only by shaping the microbiome but also by reprogramming top-down regulatory systems, including the HPA axis, circadian clock, and vagal signalling, that in turn feedback onto gut–immune–microbial dynamics. Recognising this bidirectionality strengthens the rationale for targeting the GBA through metabolic and behavioural interventions.

## 3. Metabolic Reprogramming and Neuroprotection

### 3.1. Mitochondrial Bioenergetics and Oxidative Stress

It is well reported that mitochondria are central to energy metabolism and redox homeostasis. Dysfunctional mitochondrial bioenergetics, characterised by impaired oxidative phosphorylation and excessive reactive oxygen species (ROS) production, mainly contribute to neurodegeneration, metabolic disorders, and ageing. Strategies that enhance mitochondrial function and reduce oxidative stress are thus vital for neuroprotection and metabolic health. Thus, IF, by promoting metabolic switching from glucose to lipid and ketone-based substrates may enhance mitochondrial efficiency and antioxidant capacity.

Mitochondrial dysfunction is remarked early in neurodegenerative diseases such as AD, PD, and cerebral small vessel disease (CSVD). These conditions are frequently demonstrated by heightened oxidative stress and decreased adenosine triphosphate (ATP) production [60,61,62]. For example, mitochondrial fragmentation and suppressed electron transport chain activity occur in early AD and correlate with synaptic dysfunction and cognitive decline [60]. Similarly, in CSVD, endothelial mitochondrial dysfunction leads to compromised cerebral blood flow and white matter lesions [61]. On the other hand, the GBA impacts mitochondrial dynamics through microbial metabolites, such as SCFA (i.e., butyrate), which promote mitochondrial biogenesis and reduce inflammation via PGC-1α and sirtuin activation [7]. A preclinical study using germ-free mice reported that mitochondrial-associated gene mutation in the PD’s brain, which is partially restored upon microbiota reconstitution [62]. More importantly, IF induces a metabolic switch from glucose utilisation to lipid oxidation and ketogenesis, leading to the production of ketone bodies such as β-hydroxybutyrate (BHB), hence significantly enhancing mitochondrial function [8].

BHB serves not only as an alternative energy substrate but also as a signalling molecule that affects epigenetics, inflammation, and oxidative stress whereby these metabolic adaptations improve mitochondrial respiration, reduce oxidative stress, and enhance neuronal resilience [63]. In addition, BHB also exerts versatile neuroprotective effects through its modulation of mitochondrial function, antioxidant defences, and GBA. It enhances mitochondrial respiration and ATP production by entering directly into the tricarboxylic acid cycle, bypassing glycolysis. BHB also upregulates key regulators of mitochondrial biogenesis, such as PGC-1α and mitochondrial transcription factor A (TFAM), primarily through sirtuin activation [64]. Additionally, it activates the nuclear factor erythroid 2–related factor 2 (Nrf2) pathway and inhibits HDAC, promoting the expression of antioxidant enzymes including superoxide dismutase (SOD2), catalase (CAT), and heme oxygenase-1 (HO-1), which help maintain redox homeostasis [65].

**Figure 2:**
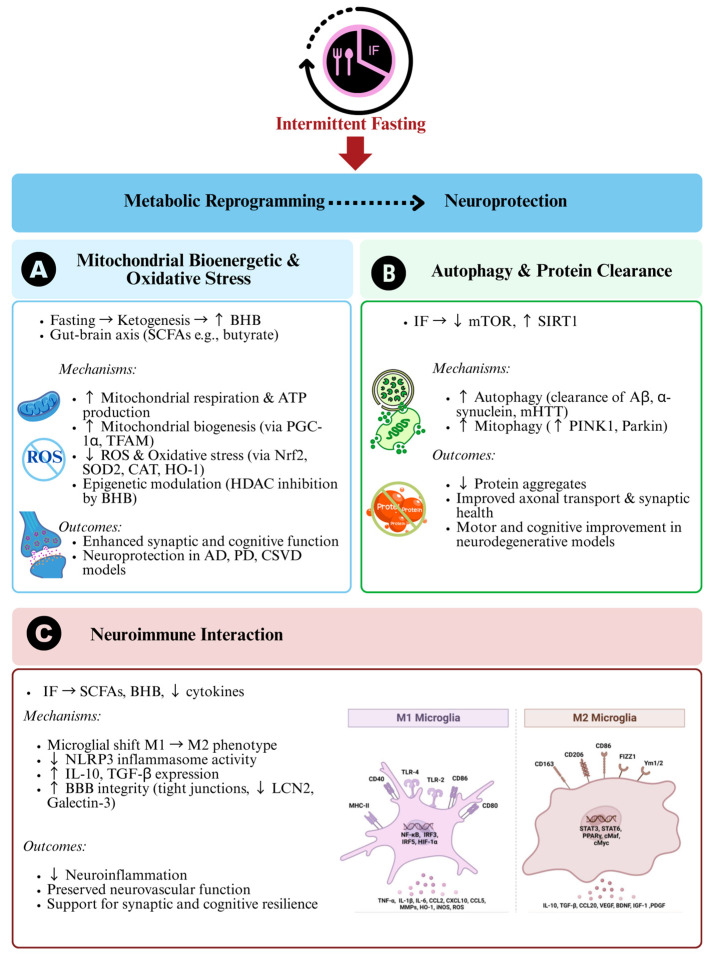
Neuroprotective mechanisms of intermittent fasting (IF) via metabolic reprogramming. This figure summarizes how IF promotes neuroprotection through three main pathways: (**A**) Mitochondrial bioenergetics and oxidative stress: IF increases β-hydroxybutyrate (BHB) and short chain fatty acids (SCFAs) (e.g., butyrate), enhancing mitochondrial respiration, biogenesis (via PGC-1α, TFAM), and antioxidant defence (via Nrf2, SOD2, CAT, HO-1), while reducing oxidative stress and supporting synaptic and cognitive function. (**B**) Autophagy and protein clearance: Through mTOR inhibition and SIRT1 activation, IF enhances autophagy and mitophagy (upregulates PINK1, Parkin), promoting clearance of toxic protein aggregates and improving neuronal health in neurodegenerative models. (**C**) Neuroimmune interaction: IF modulates microglia (M1→M2), reduces NLRP3 inflammasome activity, and strengthens blood–brain barrier integrity. These changes lower neuroinflammation and support cognitive resilience. Upward arrow (↑) indicates increase/heighten; downward arrow (↓) indicates decrease/reduce; forward arrow (→) indicates leading to/causing.

In preclinical models, BHB supplementation has been shown to reduce ROS, improve cognitive performance in AD and epilepsy, and preserve mitochondrial membrane potential [66]. Moreover, it has been demonstrated that BHB administration in a PD mouse model attenuated dopaminergic neuron loss and restored mitochondrial integrity [67]. Beyond its central effects, BHB contributes to gut health by enhancing intestinal barrier integrity via tight junction regulation and mitigating gut-derived inflammation. IF-induced elevations in BHB have also been associated with increased abundance of beneficial gut microbiota such as *Akkermansia muciniphila* and *Lactobacillus*, which are linked to improved cognitive outcomes and anti-inflammatory profiles in both animal and human studies [68] (see Figure 2).

In clinical studies, ketogenic diets or exogenous BHB supplementation have been reported to increase mitochondrial function and reduce oxidative stress markers in patients with mild cognitive impairment and metabolic syndrome [69,70]. Additionally, time-restricted eating in older adults elevated circulating BHB and improved memory and mitochondrial oxidative capacity [71]. A recent trial in individuals with type-2 diabetes showed that BHB levels were associated with improved glycaemic control, diminished 8-isoprostane (an oxidative stress marker), and improved mitochondrial respiration in peripheral blood mononuclear cells [12].

Therefore, the merging of IF, the GBA, and BHB provides a compelling framework for improving mitochondrial bioenergetics and reducing oxidative stress. Through gut-derived signals and fasting-induced ketogenesis, particularly BHB, the brain is afforded both metabolic fuel and molecular protection. These pathways offer promising therapeutic targets for neurodegenerative disorders and metabolic diseases.

### 3.2. Autophagy and Protein Clearance

Autophagy is a conserved lysosomal degradation pathway that is essential for the clearance of misfolded proteins and damaged organelles. Impaired autophagy contributes to the accumulation of neurotoxic aggregates such as amyloid-β (Aβ), α-synuclein, and mutant huntingtin (mHTT) proteins [72]. IF has been shown to robustly activate autophagy through inhibition of mechanistic target of rapamycin (mTOR) and activation of SIRT1 [13,73], both of which are responsive to nutrient depletion and SCFA/BHB signalling. These effects are systemically coordinated and may be enhanced through GBA modulation. SCFAs like butyrate and propionate have been shown to influence epigenetic regulation of autophagy genes via HDAC inhibition [25].

In AD models, IF reduces amyloid plaque burden and tau hyperphosphorylation, in part by enhancing autophagic flux [46,74]. Similar effects have been observed in PD models, where IF promotes clearance of α-synuclein aggregates and preserves dopaminergic neurons [75]. In addition, autophagy induction also improves axonal transport and synaptic function, suggesting benefits beyond proteostasis [76].

Moreover, IF-related autophagy may synergise with mitophagy, a selective form of autophagy targeting damaged mitochondria. Enhanced mitophagy improves mitochondrial turnover and limits oxidative stress, thereby creating a neuroprotective feedback loop [77]. A recent literature has highlighted that alternate-day fasting in HD mice restored mitophagy markers such as PTEN-induced kinase 1 (PINK1) and Parkin, and attenuated motor deficits [78]. Importantly, gut microbiota may amplify IF-induced autophagy via metabolite-mediated modulation of mTOR pathways. This suggests a bidirectional interplay where fasting and microbiota co-regulate lysosomal clearance mechanisms critical for neurodegeneration prevention.

### 3.3. Neuroimmune Interactions

Apart from the above-mentioned metabolic reprogramming and neuroprotective role of IF, the brain is widely acknowledged as an immunologically dynamic organ. For example, microglia, the resident immune cells of the CNS, exist along a spectrum from pro-inflammatory (M1) to anti-inflammatory (M2) phenotypes. Interestingly, IF has been shown to promote M2 polarisation, reduce neuroinflammatory tone, and preserve synaptic microenvironments, thereby reducing chronic neuroinflammation [79].

These immunomodulatory effects are partially mediated by the gut microbiota, including SCFA signalling (particularly butyrate and acetate), reduced systemic cytokine load, and ketone-induced suppression of the NLRP3 inflammasome [80]. BHB inhibits NLRP3 activation via modulation of potassium efflux and ROS production [81]. Furthermore, preclinical studies using an obese mouse model indicated that fasting upregulates the expression of neuroimmune modulators such as IL-10 and transforming growth factor beta (TGF-β), which contribute to tissue repair and synaptic stability [82].

Recent findings also implicate IF in modulating glial–neuronal interactions and blood–brain barrier integrity. In a recent pre-clinical study, IF preserved tight junction protein expression in the hippocampal vasculature of high-fat diet-fed mice by downregulating glial cell activator and pro-inflammatory mediators such as lipocalin-2 and galectin-3, respectively, in which they may further exacerbate neurodegenerative diseases if left untreated [83], suggesting a role in maintaining neurovascular health, a critical aspect often disrupted in early neurodegeneration. In summary, IF orchestrates neuroimmune homeostasis through GBA-integrated signals that regulate cytokine networks, glial activity, and immune–metabolic resilience. These immunological adaptations are critical for long-term neuroprotection and cognitive preservation. Figure 2 illustrates the neuroprotective mechanisms of IF via metabolic reprogramming.

## 4. Disease-Specific Evidence

Neurodegenerative disorders are characterised by progressive neuronal loss, oxidative stress, protein aggregation, chronic inflammation, and impaired cellular clearance mechanisms. These disorders often overlap mechanistically, particularly at the level of neuroimmune dysregulation, mitochondrial dysfunction, and barrier breakdown all of which are influenced by GBA signalling and modifiable IF. Building on it is proposed that IF exerts neuroprotective effects through an integrated set of mechanisms: (i) remodelling of gut microbiota toward SCFA-producing and anti-inflammatory taxa; (ii) enhancement of mitochondrial bioenergetics and antioxidant defences (via BHB, SCFAs, SIRT1); (iii) activation of autophagy and mitophagy through mTOR inhibition and microbial metabolite signalling; (iv) entrainment of circadian clocks that regulate neuroimmune tone and proteostasis; and (v) attenuation of systemic and central inflammation via reduced endotoxemia and microglial reprogramming. These mechanisms act in parallel to influence disease-specific trajectories.

### 4.1. Alzheimer’s Disease (AD)

AD, the leading cause of dementia worldwide, is characterised by extracellular Aβ accumulation, tau hyperphosphorylation, neuroinflammation, and synaptic failure. Increasing evidence links gut dysbiosis to cognitive decline through mechanisms including BBB disruption, LPS-induced inflammation, and reduced SCFA availability [73]. Preclinical studies show that IF enhances hippocampal BDNF expression, reduces Aβ load, and preserves cognitive function in models such as 3xTg-AD and APP/PS1 mice [74,75]. These effects are accompanied by increased abundance of *Akkermansia muciniphila* and *Bifidobacterium* spp., improved gut barrier integrity, and higher SCFA levels.

Moreover, IF has been demonstrated to serve as a disease-modifying agent in preclinical AD models, remarkably through enhancing autophagic flux and mitophagy, key processes often impaired in AD brains [46,74]. Particularly, alternate-day and time-restricted feeding schemes in AD mice reduce hippocampal Aβ deposition [84], improve spatial memory [47], and elevate BDNF expression [85], an effect linked to increased activation of the PGC-1α/NRF1/TFAM axis that governs mitochondrial biogenesis [58,86]. Moreover, current evidence also suggests that IF may also reprogram glial metabolism. A 2022 study demonstrated that IF can modify the microglial transcriptomes toward a homeostatic phenotype, suppressing NLRP3 inflammasome activity and shifting neuroinflammatory cytokine output from IL-1β to IL-10 [87]. This transition may be paramount in restoring synaptic pruning and cognitive resilience.

Importantly, human data, though emerging, are beginning to align. A 2019 pilot study [88] involving elderly participants sticking to a 16:8 time-restricted eating schedule reported not only cognitive gains on the Montreal cognitive assessment (MoCA) but also reduced peripheral IL-6, a biomarker of systemic inflammation. Supplementing these findings, neuroimaging (i.e., MRI) analyses from a 12-week fasting study in mild cognitive impairment (MCI) patients revealed increased hippocampal volume and enhanced functional connectivity within the default mode network changes that may reflect neurovascular remodelling and synaptic preservation [89].

Additionally, mitochondrial markers such as PGC-1α and SIRT1 are upregulated under IF, promoting neuronal resilience and metabolic flexibility. Concurrent autophagic activation aids in Aβ clearance and tau degradation. These benefits may be amplified through circadian alignment, which restores sleep architecture and glymphatic clearance, key components in AD pathophysiology [76,77,78].

Notably, these human imaging signatures may serve as early biomarkers of IF efficacy, deserving further research into the interplay between circadian metabolism and related microbiome [90], glymphatic clearance [91,92], and neurodegeneration. Future studies ought to deliberate on integrating cerebrospinal fluid (CSF) biomarkers, tau-positron emission tomography (PET) imaging, and chrononutrition profiling to fine-tune the therapeutic windows.

### 4.2. Parkinson’s Disease (PD)

PD is pathologically defined by dopaminergic neuronal loss in the substantia nigra pars compacta and cytoplasmic α-synuclein inclusions. Beyond dopamine-centric mechanisms, PD is increasingly recognised as a metabolic disorder of neuronal energetics [93]. In this context, IF correspond to a bioenergetic intervention that modulates mitochondrial dynamics and proteostatic burden.

Pre-clinical animal studies demonstrated that alternate-day fasting protects dopaminergic circuitry and improves motor phenotypes, an effect partly mediated by adenosine monophosphate-activated protein kinase (AMPK) activation and mitophagy induction [75]. BHB, a ketone body increased during fasting, upregulates SIRT3 in midbrain neurons, inhibits pyroptosis by downregulating signal transducer and activator of transcription 3 (STAT3)-mediated NLRP3 inflammasome activation for PD models in vivo and in vitro, improving mitochondrial antioxidant defences, and reducing α-synuclein oligomerisation [94,95].

Furthermore, IF-induced gut microbial shifts, particularly the enrichment of SCFA-producing taxa such as *Faecalibacterium prausnitzii*, may indirectly modulate central dopaminergic tone via microbiota–gut–brain signalling pathways [96,97]. SCFAs have been shown to cross the blood–brain barrier and enhance dopaminergic vesicle packaging, possibly through G-protein coupled receptor 41/43 (GPR41/43) activation and downstream modulation of tyrosine hydroxylase expression [98].

While clinical evidence remains mostly observational, preliminary epidemiological data recommend that habitual IF or Ramadan-style fasting shows a relationship with slower PD progression and better motor scores [99,100]. However, thorough trials are needed. Future directions ought to incorporate wearable motor sensors, neuroimaging of nigrostriatal integrity (e.g., dopamine transporter single photon emission computed tomography, DAT-SPECT) [101], and CSF metabolomics to capture early IF-responsive biomarkers.

### 4.3. Huntington’s Disease (HD)

HD, caused by expanded CAG repeats in the HTT gene, results in progressive motor, cognitive, and psychiatric dysfunction. The mHTT protein forms toxic aggregates that disrupt autophagy and axonal transport. IF offers a rare example of a non-pharmacologic intervention that directly modulates pathogenic protein turnover. In HD models, IF induces transcriptional programs via Forkhead Box O3 (FOXO3) and TFEB, restoring lysosomal acidification and enhancing clearance of mHTT aggregates [102,103].

Moreover, translational barriers remain significant. HD patients often experience unintended weight loss and hypermetabolism, complicating IF implementation [104]. However, metabolically-informed IF protocols such as cyclic ketogenic TRE with amino acid supplementation may address these challenges while preserving neuroprotective effects [105]. Emerging human trials are exploring TRE’s impact on executive function and metabolic resilience in prodromal HD [106]. These studies could pioneer a personalised, stage-specific nutritional approach to neurodegeneration. Novel endpoints should include neural entropy measures from electroencephalography (EEG), digital phenotyping via smartphone-based motor tracking, and longitudinal microbiome–brain axis profiling.

### 4.4. Amyotrophic Lateral Sclerosis (ALS)

ALS remains among the most therapeutically refractory neurodegenerative diseases. Characterised by rapid degeneration of upper and lower motor neurons, ALS pathogenesis involves a toxic triad of oxidative stress, mitochondrial dysfunction, and neuroinflammation, which are directly modulated by IF.

While direct studies on IF in SOD1-G93A mice are limited, related interventions have shown promising results. For instance, trimetazidine treatment preserved neuromuscular junction integrity in SOD1-G93A mice, suggesting that metabolic interventions can benefit neuromuscular junction health in ALS models [107]. Mechanistically, IF boosts PINK1/Parkin-mediated mitophagy and reduces cytosolic ROS via Nrf2 pathway activation [108]. Importantly, fasting also remodels astrocytic metabolism, decreasing glutamate excitotoxicity and increasing lactate shuttling to motor neurons [109].

Patients with ALS often experience weight loss and hypermetabolism, complicating the implementation of IF. However, a case study demonstrated that a time-restricted ketogenic diet could be safely implemented in an ALS patient, leading to improvements in various health parameters [110]. Moreover, exogenous ketogenic supplements, such as ketone esters and medium-chain triglycerides, have been shown to mitigate ageing processes and may help preserve muscle mass while mimicking fasting physiology [111]. Despite limited clinical data, there is a compelling rationale for early-phase trials incorporating bioenergetic phenotyping, motor unit number estimation (MUNE), and CSF neurofilament light chain (NfL) tracking as endpoints [112]. Additionally, investigating IF’s role in modifying neuroimmune profiles, particularly T-cell infiltration and microglial polarisation, could yield novel therapeutic insights [113].

In conclusion, across diverse neurodegenerative disorders, IF potentially exerts convergent protective effects via autophagy induction, mitochondrial optimisation, neuroimmune modulation, and systemic metabolic recalibration. Yet, the field faces key knowledge gaps: the heterogeneity of IF protocols, interindividual variability in metabolic responses, and the absence of robust clinical biomarkers. A coordinated, transdisciplinary effort is required to elucidate the neurobiological time-course of IF effects and to develop patient-tailored fasting interventions. Future work should prioritise multimodal biomarker development, leveraging integrative omics, advanced neuroimaging, and digital phenotyping. Only through such systems-level approaches can IF transition from a promising intervention to a precision neurotherapeutic platform. Table 2 summarises the clinical and preclinical studies on IF in neurodegenerative diseases.

## 5. Clinical Translation and Future Directions

As discussed, IF has transitioned from a metabolic intervention to a neuromodulator paradigm with the potential to influence the course of neurodegenerative diseases. Yet, translating its promise into clinical practice demands a rigorous interrogation of safety, personalisation, mechanistic monitoring, and ethical deployment. The next frontier lies not in whether IF works, but in how, for whom, and under what systems-level conditions can be safely and effectively implemented.

### 5.1. Safety, Adherence, and Ethical Considerations

While preclinical data overwhelmingly support IF’s neuroprotective effects, clinical translation remains constrained by physiological, behavioural, and ethical considerations, particularly in vulnerable populations such as older adults, patients with frailty, or those with cognitive impairment. Risks such as hypoglycaemia, sarcopenia, dehydration, and micronutrient deficiencies are not trivial and may be exacerbated by comorbidities or polypharmacy [115].

Importantly, neurodegenerative patients frequently show altered energy metabolism, disturbed circadian rhythms, and impaired appetite regulation, which may interact unpredictably with fasting regimens [116]. Therefore, IF cannot be a “one-size-fits-all” strategy. A tiered risk stratification system, perhaps incorporating frailty indices [117], bone mineral content (e.g., dual-energy X-ray absorptiometry, DXA scans) [118], and metabolic resilience testing, is somewhat necessary before clinical implementation.

In addition, adherence is another critical barrier, whereby cognitive decline impairs executive function and routine maintenance, making unsupervised IF possibly dangerous [119]. Thus, digital health solutions including app-guided timers, metabolic feedback systems (e.g., ketone biosensors) [120], and caregiver-linked compliance platforms may possibly bridge this gap. Additionally, machine learning algorithms integrated into wearables may suggest dynamic monitoring of fasting-related physiology, flagging early signs of risk or non-compliance.

Furthermore, ethical considerations must not be overlooked. As enthusiasm grows, so too does the risk of promoting restrictive eating behaviours in cognitively vulnerable populations. Clinicians must balance enthusiasm with caution and equity, ensuring that IF interventions do not inadvertently widen health disparities due to access issues, technological divides, or socioeconomic factors.

### 5.2. Precision Nutrition: Toward Biomarker-Guided, Individualized Fasting

A paradigm shift is underway from standardised IF protocols to precision fasting guided by biological individuality. This transformation is underpinned by growing evidence that metabolic, genetic, epigenetic, and microbiome-based factors dictate individual responses to fasting. For example, polymorphisms in genes such as SIRT1, FOXO3, and MTOR may influence autophagy and oxidative stress thresholds, affecting neuroprotective outcomes [102,103].

Metabolomic signatures, particularly fasting-induced changes in circulating ketones (e.g., BHB), lactate, acylcarnitine, and branched-chain amino acids, could serve as real-time biomarkers of bioenergetic shifts [112]. At the same time, gut microbial profiles such as the abundance of butyrate-producing species (*Faecalibacterium, Roseburia*) may predict responses to IF via their role in maintaining GBA integrity and modulating neuroinflammation [32,96,97]. Emerging computational models can integrate these datasets to identify “fasting responders” vs. “non-responders.” In addition, artificial intelligence (AI)-driven personalisation engines may soon be able to prescribe IF windows dynamically, optimising fasting durations, feeding windows, and nutrient composition based on predicted neuroprotective efficacy. Such approaches would move IF from a behavioural recommendation to a precision neurotherapeutic protocol.

Notably, integration with circadian biomarkers such as melatonin rhythm, cortisol amplitude, and sleep phase could enable chrono nutritional personalisation [121,122]. This could be particularly impactful for neurodegenerative patients, who often suffer from disrupted circadian biology. Personalised circadian aligned IF may enhance both adherence and efficacy.

### 5.3. Synergistic Therapeutic Combinations: A Systems-Level Strategy

Given the multifactorial nature of neurodegeneration, mono-therapeutic approaches are unlikely to yield durable clinical benefits. IF’s pleiotropic effects make it an ideal backbone for multi-modal therapeutic synergies. For example, exercise is a natural co-intervention, sharing overlapping mechanisms with IF, including BDNF upregulation, mitochondrial biogenesis, and epigenetic reprogramming. Co-administration of IF and aerobic or resistance training has shown additive neurocognitive benefits in preclinical models and pilot clinical trials [123,124].

Secondly, probiotics and psychobiotics may further enhance IF-induced neuroplasticity by modulating gut microbiota composition and metabolite production [125]. For example, combining IF with *Lactobacillus plantarum* or *Bifidobacterium longum* has been shown to synergistically reduce neuroinflammatory markers in mouse models [126,127]. Next is the use of pharmacological enhancers, such as low-dose rapamycin (an mTOR inhibitor) or metformin, which may potentiate IF-induced autophagy, while simultaneously correcting metabolic dysregulation in high-risk patients [128]. However, timing and dosing must be carefully calibrated to avoid adverse effects or pathway overstimulation.

Furthermore, dietary quality during feeding windows is also critical. Anti-inflammatory, ketogenic, or Mediterranean-inspired meal compositions can amplify IF’s neuroprotective effects [70]. These diets not only support metabolic goals but may modulate gut microbial resilience, further promoting CNS homeostasis. Finally, cognitive interventions such as memory training, mindfulness, or non-invasive brain stimulation may act synergistically with IF by enhancing neurogenesis and synaptic remodelling. Such integrative strategies reflect the necessity of treating neurodegeneration not as a single-pathway disorder, but as a systems failure requiring systems-level solutions. Table 3 summarises the proposed personalised fasting strategies and their theoretical applications based on patient profiles. We highlighted a specific patient profile for a recommended IF strategy, potential risks, mechanistic rationale, and suggested biomarkers for monitoring reflecting a precision medicine approach.

IF is no longer merely a metabolic intervention; it is an evolving neurotherapeutic platform. Thus, it is crucial to reframe IF as a precision neurotherapeutic. As clinical translation advances, the key will be to integrate IF into a broader precision medicine framework, guided by multi-omics biomarkers, digital health tools, and synergistic therapies. Moving forward, randomised controlled trials must embrace stratified design, longitudinal biomarker integration, and real-world adherence modelling. By embedding IF into a dynamic, personalised, and ethically grounded framework, we can begin to reshape the therapeutic landscape of neurodegeneration from managing decline to preserving resilience.

## 6. Conclusions

IF is emerging as a powerful neurometabolic intervention that transcends caloric restriction, targeting the complex pathophysiology of neurodegenerative diseases through modulation of the GBA, induction of autophagy, circadian realignment, and suppression of neuroinflammation. Preclinical studies across AD, PD, HD, and ALS models demonstrate consistent benefits in synaptic integrity, neurotrophic signalling, and proteostasis, mediated by regulators such as BDNF, SIRT1, mTOR, and gut-derived metabolites like SCFAs and BHB. Early human trials, though limited, reveal promising improvements in cognition, neuroplasticity, and inflammatory biomarkers. To advance clinical translation, IF must be redefined as a precision neurotherapeutic personalised, integrable, and guided by genomic, metabolic, and microbiomics profiles. Rigorous, stratified trials and digital adherence tools will be key to optimising efficacy while addressing safety, equity, and ethical deployment in vulnerable populations. With its unique convergence of mechanistic depth, translational potential, and scalability, IF may ultimately transform the management of neurodegenerative diseases from reactive care to proactive neuro-resilience.

## Figures and Tables

**Table 1:** Intermittent fasting (IF) alters gut–brain inflammatory mediators modulating neurodegenerative signalling.

Mediator/Pathway	Site of Action	Effect of IF	Neurodegenerative Relevance	Ref.
TLR4	Gut epithelium Microglia	Downregulation of expression Reduced LPS-mediated signalling	Reduces microglial activation and neuroinflammation	[37,38]
NF-κB	Intestinal mucosa CNS	Suppressed activation via SCFA and SIRT1	Limits transcription of pro-inflammatory cytokines	[40]
NLRP3 Inflammasome	Peripheral macrophages Microglia	Inhibited via β-hydroxybutyrate and butyrate	Reduces IL-1β secretion and neurotoxicity	[41]
Tight junction proteins (e.g., Occludin, Claudin-1)	Gut barrier	Upregulated expression and improved barrier function	Prevents systemic inflammation via endotoxin leakage	[39]
SCFA (e.g., Butyrate, Propionate)	Gut lumen Brain	Increased production HDAC inhibition Anti-inflammatory effects	Enhances BDNF, reduces oxidative and inflammatory damage	[21,24,25]
IL-6, TNF-α, CRP	Blood Brain	Decreased circulating levels following IF	Reduced systemic-to-CNS inflammatory signalling	[42,43]
AhR	Gut immune cells Brain	Activated by microbial indoles under IF	Regulates mucosal immunity and neuroimmune crosstalk	[48]

AhR, Aryl hydrocarbon receptor; BDNF, brain-derived neurotrophic factor; CNS, central nervous system; CRP, C-reactive protein; HDAC, histone deacetylase; IL, interleukins; LPS, lipopolysaccharides; NF-κB, nuclear factor kappa B; NLRP3, NOD-, LRR- and pyrin domain-containing protein 3; SCFA, short chain fatty acids; SIRT1, sirtuin 1; TLR4, Toll-like receptor 4; TNF-α, tumour necrosis factor alpha.

**Table 2:** Summary of clinical and preclinical studies on intermittent fasting in neurodegenerative diseases.

Disease	Study Type	Model/Population	IF Protocol	Key Findings
Alzheimer’s Disease (AD)	Preclinical [29]	3xTg-AD mice	ADF for 3 months	↓ neuronal loss, Aβ oligomers and tau hyperphosphorylation. ↑ cognitive function. ↑ hippocampal synaptic plasticity and BDNF. ↑ mitochondrial bioenergetic function.
	Clinical [88]	Elderly individuals with subjective cognitive decline	16:8 TRE for 12 weeks	↑ MoCA scores ↓ plasma IL-6
	Clinical [89]	MCI patients	16:8 TRE for 12 weeks	↑ Hippocampal volume ↑ DMN connectivity
Parkinson’s Disease (PD)	Preclinical [114]	MPTP-induced PD mice	fasting mimicking diet (FMD), fasting 3 days followed by 4 days of refeeding for three 1-week cycles	↓ Nigral cell loss ↑ motor behaviour Transplantation of faecal microbiota, increased dopamine levels
	Clinical [99,100]	PD patients practising Ramadan fasting	~14-h daily fast for 30 days	↓ UPDRS motor scores ↑ sleep quality
Huntington’s Disease (HD)	Preclinical [102,103]	R6/2 transgenic mice	ADF for 8 weeks	↓ mHTT aggregation ↑ motor function
	Clinical [106]	Prodromal HD (NCT06490367)	TRE (10-h feeding) for 12 weeks	Pending (sleep quality, physical activity, mood, dietary composition, and mitochondrial function)
Amyotrophic Lateral Sclerosis (ALS)	Preclinical [107]	SOD1-G93A transgenic mice	ADF starting pre-symptomatically	↑ Lifespan (~13%) ↑ neuromuscular strength
	Clinical [110]	ALS patients attempting modified fasting	IF with nutritional support	Mixed tolerance anecdotal reports of subjective benefit

Notes, ↓ represent decrease/reduce; ↑ represent increase/improved. Aβ, amyloid beta; ADF, alternate day fasting; BDNF, brain-derived neurotrophic factor; DMN, default mode network; IL-6, Interleukin-6; MCI, mild cognitive impairment; MoCA, Montreal cognitive assessment; MPTP, 1-Methyl-4-phenyl-1,2,3,6-tetrahydropyridine; SOD1-G93A, superoxide dismutase 1-strain G93A; TRE, time-restricted eating; UPDRS, Unified Parkinson’s disease rating scale.

**Table 3:** Putative personalised fasting strategies and their theoretical applications based on patient profiles.

Patient Profile	Suggested IF Strategy	Potential Risks	Mechanistic Rationale	Monitoring Biomarkers
Elderly with mild cognitive impairment	12:12 progressing to 14:10 TRF	Sarcopenia, malnutrition Cognitive decline	Circadian rhythm entrainment Enhanced BDNF expression Reduced neuroinflammation	MoCA scores IL-6 IGF-1 Bone density (DXA)
Parkinson’s disease (early-stage)	16:8 TRF or alternate day fasting	Hypoglycaemia Medication timing conflicts	Autophagy activation SIRT1-mediated dopaminergic preservation Mitochondrial biogenesis	UPDRS BHB SIRT1 expression oxidative stress markers
Genetic risk carriers (e.g., APOE4+)	14:10 TRF with Mediterranean meals	Lipid metabolism sensitivity	Reduction of amyloidogenic processing Modulation of cholesterol metabolism	Plasma Aβ42/40 Lipid panel Ketone levels
Metabolically obese with neuroinflammation	Alternate-day or 5:2 fasting	Adherence Binge eating during feeding windows	Reduction of systemic and CNS inflammation Insulin sensitisation	CRP, IL-1β, fasting insulin Gut microbial diversity (16S rRNA)
ALS with weight loss risk	12:12 mild TRF with caloric support	Cachexia, catabolism Nutrient deficiencies	Mild mitophagy induction ROS reduction without exacerbating muscle loss	Weight/BMI trajectory, creatinine, cytokine profiles
Circadian misaligned (e.g., night-shift workers or AD)	Chronotype-adjusted 10:14 TRF	Disrupted sleep Metabolic misalignment	Alignment of feeding-fasting cycles to circadian biology Melatonin-cortisol axis stabilization	Cortisol rhythm Melatonin, actigraphy-derived sleep quality
HD patients (early symptomatic)	14:10 TRF with high-protein support	Metabolic dysregulation Weight instability	Improved autophagy-lysosome function Suppression of mHTT aggregation	Motor scale, mHTT levels, amino acid profile
High-performing adults seeking neuroprotection	16:8 TRF with exercise pairing	Over-restriction Overtraining	Enhanced neurogenesis BDNF synergism with physical activity	BHB lactate VO2max Cognitive flexibility tests

BDNF, brain-derived neurotrophic factor; BHB, beta-hydroxybutyrate; CNS, central nervous system; CRP, C-reactive protein; IGF-1, Insulin-like growth factor 1; mHTT, mutant huntingtin protein; MoCA, Montreal cognitive assessment; TRF, time-restricted feeding; UPDRS, unified Parkinson’s Disease rating scale.

## Abbreviations

The following abbreviations are used in this manuscript:

AD	Alzheimer’s disease
AhR	Aryl hydrocarbon receptor
ALS	Amyotrophic lateral sclerosis
AMPK	Adenosine monophosphate-activated protein kinase
ATP	Adenosine triphosphate
Aβ	Amyloid-β
BDNF	Brain-derived neurotrophic factor
BHB	β-hydroxybutyrate
CAT	Catalase
CNS	Central nervous system
CRP	C-reactive protein
CSF	Cerebrospinal fluid
CSVD	Cerebral small vessel disease
DAT-SPECT	Dopamine transporter single photon emission computed tomography
DMN	Default mode network
DXA	Dual-energy X-ray absorptiometry
EEG	Electroencephalography
FMD	Fasting-mimicking diet
FOXO3	Forkhead Box O3
GBA	Gut–brain axis
HD	Huntington’s disease
HDAC	Histone deacetylase
HO-1	Heme oxygenase-1
IF	Intermittent fasting
IGF-1	Insulin-like growth factor 1
IL	Interleukins
iNOS	Inducible nitric oxide synthase
LPS	lipopolysaccharides
MCI	Mild cognitive impairment
mHTT	Mutant huntingtin
MoCA	Montreal cognitive assessment
MPTP	1-Methyl-4-phenyl-1,2,3,6-tetrahydropyridine
mTOR	Mechanistic target of rapamycin
MUNE	Motor unit number estimation
NfL	Neurofilament light chain
NF-κB	Nuclear factor-kappa B
NLRP3	NOD-, LRR- and pyrin domain-containing protein 3
Nrf2	Nuclear factor erythroid 2–related factor 2
PD	Parkinson’s disease
PET	Positron emission tomography
PGC-1α	Peroxisome proliferator-activated receptor-γ coactivator 1-α
PINK1	PTEN-induced kinase 1
ROS	Reactive oxygen species
SCFA	Short-chain fatty acids
SIRT1	Sirtuin
SOD	superoxide dismutase
STAT3	Signal transducer and activator of transcription 3
TFAM	Mitochondrial transcription factor A
TGF-β	Transforming growth factor beta
TLR4	Toll-like receptor 4
TNF-α	Tumour necrosis factor alpha
UPDRS	Unified Parkinson’s disease rating scale
